# Use of oral glucocorticoids and risk of skin cancer and non-Hodgkin's lymphoma: a population-based case–control study

**DOI:** 10.1038/sj.bjc.6604796

**Published:** 2008-11-25

**Authors:** A Ø Jensen, H F Thomsen, M C Engebjerg, A B Olesen, S Friis, M R Karagas, H T Sørensen

**Affiliations:** 1 Department of Clinical Epidemiology, Aarhus University Hospital, Aarhus C, Denmark; 2 Department of Dermatology, Aarhus University Hospital, Aarhus C, Denmark; 3 Institute of Cancer Epidemiology, Danish Cancer Society, Copenhagen, Denmark; 4 Department of Community and Family Medicine, Section of Biostatistics and Epidemiology, Dartmouth Medical School, Lebanon, NH, USA

**Keywords:** oral glucocorticoids, non-Hodgkin's lymphoma, skin cancer, case–control study, epidemiology

## Abstract

In North Jutland County, Denmark, we investigated whether use of oral glucocorticoids was associated with an increased risk of developing basal cell carcinoma (BCC), squamous cell carcinoma (SCC), malignant melanoma (MM), and non-Hodgkin's lymphoma (NHL). From the Danish Cancer Registry we identified 5422 BCC, 935 SCC, 983 MM, and 481 NHL cases during 1989–2003. Using risk-set sampling we selected four age- and gender-matched population controls for each case from the Civil Registration System. Prescriptions for oral glucocorticoids before diagnosis were obtained from the Prescription Database of North Jutland County on the basis of National Health Service data. We used conditional logistic regression to estimate incidence rate ratios (IRRs), adjusting for chronic medical diseases (information about these were obtained from the National Patient Registry) and use of other immunosuppressants. We found slightly elevated risk estimates for BCC (IRR, 1.15 (95% CI: 1.07–1.25)), SCC (IRR, 1.14 (95% CI: 0.94–1.39)), MM (IRR, 1.15 (95% CI: 0.94–1.41), and NHL (IRR, 1.11 (95% CI: 0.85–1.46)) among users of oral glucocorticoids. Our study supports an overall association between glucocorticoid use and risk of BCC that cannot be explained by the presence of chronic diseases or concomitant use of other immunosuppressants.

Glucocorticoids were introduced in clinical practice in 1949 for the treatment of rheumatoid arthritis (Le Vay and Loxton, 1949), since their indications had expanded, and they are now used in many specialties (Zoorob and Cender, 1998). Glucocorticoids exert immunosuppressive effects, and there has been concern that their use may promote immune-related cancers, such as basal cell carcinoma (BCC), squamous cell carcinoma (SCC), malignant melanoma (MM), and non-Hodgkin's lymphoma (NHL). Despite the extensive use of glucocorticoids and their carcinogenic potential, epidemiological data are sparse. Two recent studies conducted in Denmark and the USA reported increased risks of skin cancer and NHL among users of systemic glucocorticoids who were not transplant recipients. In both studies, use of glucocorticoids was associated with a two-fold increased risk of SCC and NHL, and a modestly increased risk of BCC (Karagas *et al*, 2001; Sorensen *et al*, 2004). However, two recent Swedish studies have questioned whether these associations were causal (Askling *et al*, 2005; Baecklund *et al*, 2006). The first study found that patients with polymyalgia rheumatica and giant cell arteritis did not have an increased risk of lymphoma, although these patients were heavy users of glucocorticoids (Askling *et al*, 2005). The second study reported that the risk of lymphomas was associated with activity of rheumatoid arthritis, rather than treatment with glucocorticoids (Baecklund *et al*, 2006). Thus, it remains unclear whether the increased risk of immune-related cancers stems from the inflammatory activity of the chronic autoimmune diseases being treated, other immunosuppressive drugs used to treat these conditions, or the glucocorticoids *per se*.

To address these questions, we conducted a large population-based case–control study in Denmark. We examined whether oral glucocorticoid use was associated with an increased risk of skin cancers and NHL, considering the chronic diseases being treated and use of other immunosuppressive drugs.

## Materials and methods

We conducted this population-based case–control study in North Jutland County, with a population of about 500 000 inhabitants (approximately 9% of the Danish population) (Sorensen *et al*, 2008). Use of the unique 10-digit civil registry number, assigned to all Danish residents (Frank, 2000), allowed us to link data on cancer incidence, drug prescriptions, and hospital in- and outpatient diagnoses.

From the Danish Cancer Registry (DCR), we identified all patients registered with a diagnosis of skin cancer or NHL in North Jutland County during 1989–2003. The DCR, established in 1943, records primary cases of cancer on a nationwide basis with a high degree of accuracy and completeness (Storm *et al*, 1997). Data in the DCR include cancer type, site, morphology, and date of diagnosis. Tumours are coded according to a modified version of the seventh revision of the International Classification of Diseases (ICD-7). Since 1978, tumours have also been classified according to the first version of the International Classification of Diseases for Oncology (ICD-O-1), which includes a four-digit code for tumour morphology.

We used ICD-7 codes 1910–1919 to identify all BCC and SCC cases registered in North Jutland County during 1989–2003. For MM we used ICD-7 codes 1900–1909, and for NHL we used ICD-7 codes 2000–2009 and 2020–2029. We excluded patients with cancer diagnosis or organ transplantation before the skin cancer or NHL diagnosis. For BCC we included only patients with ICD-O-1 morphology codes 80903, 80913, 80923, 80933, and 81233. For SCC we included only patients with ICD-O-1 codes 80513, 80703, 80713, 80743, 80763, 80943, and 80953. We identified 5422 BCC, 935 SCC, 983 MM, and 481 NHL cases.

The Civil Registration System contains information about the vital status, date of death, and the area of residence of all Danish residents, and is updated daily. Using the Civil Registration System, we selected four population controls for each case. Cases and controls were individually matched by age and gender on the basis of risk-set sampling (Wacholder *et al*, 1992); that is, the controls were sampled among county residents who were at risk of a first skin cancer or NHL at the time the corresponding case was diagnosed (index date assigned to controls). We excluded controls with cancer diagnosis or organ transplantation before the index date. A total of 29 664 controls were identified. Using risk-set sampling, the estimated exposure odds ratio in a case–control design is an unbiased estimate of the incidence rate ratio (IRR) in a corresponding cohort study (Szklo and Nieto, 2000).

Data on oral glucocorticoid prescriptions were obtained from the Prescription Database of North Jutland County, a research database based on National Health Service data (Gaist *et al*, 1997; Nielsen *et al*, 1997). The database collects data on all prescriptions filled by ambulatory patients and forwards data on reimbursable medicines to the local regional Health Service section on a monthly basis. This Health Service, in turn, refunds 50–75% of costs. The database was established in 1989 (with complete coverage since 1991), and includes patients’ civil registry number as well as information on the type of drug prescribed (according to the Anatomical Therapeutical Chemical (ATC) classification system) (Dukes, 1993), the date when the prescription was filled, packaging size, the number of pills in each package, and the amount of drug in each pill. Indications for drug use and dosing schedules are not included. The ATC codes for glucocorticoids used in this study are listed in Appendix 1.

Individuals with certain chronic medical conditions are more likely to receive prescriptions for glucocorticoids. Some of these conditions are also associated with an elevated risk of skin cancer (Jensen *et al*, 2008) and NHL (Kinlen, 1992). To control for the potentially confounding effects of chronic medical conditions, we used the Danish National Registry of Patients to retrieve all hospital diagnoses recorded among our study population from January 1, 1977 to December 31, 2003. Diagnoses are coded according to the ICD-8 classification through 1993 and the ICD-10 thereafter. The Danish National Registry of Patients contains information on all non-psychiatric hospital admissions since 1977 (Andersen *et al*, 1999) and outpatient visits since 1995. We classified the diagnoses of chronic diseases into the four categories listed in Appendix 2. In addition, we retrieved information on prescriptions redeemed for azathioprine (ATC code: L04AX01) and methotrexate (ATC code: L01BA01), as use of these drugs has been associated with an increased risk of skin cancer (Glover *et al*, 1997; Jensen *et al*, 1999; Marcen *et al*, 2003) and NHL (Kinlen, 1992).

### Statistical analysis

For each subject, we identified all prescriptions for oral glucocorticoids before the date of primary skin cancer or NHL diagnosis, or before the corresponding index date for the matched population control. We computed the total amount of drug prescribed before the index date by multiplying the package size, the number of pills in each package, and the amount of drug in each pill. When the amount of drug could not be calculated (for instance, due to missing information in the prescription database), the average amount dispensed for that particular drug was estimated. Average amounts were applied to 13% of the identified prescriptions for oral glucocorticoids.

Initially, we constructed contingency tables for cases and controls by demographic characteristics (age and sex), anatomic site of the skin tumour, prior hospitalisations for the selected comorbid conditions, and previous prescriptions for azathioprine and methotrexate. Three models were used for the various combinations of oral glucocorticoid exposure and outcome (i.e., BCC, SCC, MM, and NHL). The first model treated the exposure as a dichotomous variable (i.e., any/no prescription for glucocorticoids before the index date). The second model assumed a linear effect of the exposure (i.e., as a continuous variable based on the estimated milligrams of prescribed glucocorticoids, with ‘no prescriptions before the index date’ as reference). The third model was fitted to test for a nonlinear effect by treating the exposure as a restricted cubic spline (Harre *et al*, 1988). Trend tests were used to evaluate the statistical significance of the effect of increasing amounts of prescribed oral glucocorticoids on the risk of these cancers (dose–response relationship). *P*-values <0.05 were considered statistically significant. We included the four chronic disease categories, listed in Appendix 2, and prescriptions for azathioprine and methotrexate as confounding factors. These variables were included in the models as dichotomous variables, that is, yes/no disease and yes/no prescription.

As cancer diagnoses might be differentially related to glucocorticoid use due to surveillance bias (Rothman, 2002), we also conducted analyses excluding prescriptions issued within one year of the skin cancer diagnosis or index date. These analyses might also point to possible effects on the duration of glucocorticoid use. We also conducted analyses stratified on the anatomic site of the skin cancers (i.e., head and neck and other sites), to evaluate the effects of glucocorticoids by level of sun exposure.

Non-reporting of diagnosed non-melanoma skin cancer (NMSC) to the DCR has been estimated to range from 12 to 40% (Frentz, 1996; Jensen *et al*, 2007). Therefore, we conducted a sensitivity analysis (Fox *et al*, 2005a; Greenland, 2005) to explore the magnitude of effects of the non-reporting of NMSC cases on our results. We expected the non-reporting to be differential between users and non-users of glucocorticoids; NMSC is rarely fatal, and therefore in severely diseased patients (such as users of glucocorticoids) clinicians could potentially deem these cancers trivial and thereby omit registration. We used the SAS-macro written by Fox, Lash and Greenland, which was adapted to perform conditional logistic regression (Fox *et al*, 2005b).

This study was approved by the Danish Protection Agency (Record no. 2004-41-4693). The statistical software packages R, version 2.4.1, and SAS, version 9.1 (SAS Institute Inc., Cary, NC), were used for all statistical analyses.

## Results

The characteristics of cancer cases and controls are presented in Table 1. The median age was 68 years among BCC, 77 years among SCC, 58 years among MM, and 64 years among NHL cases. The majority of the SCC tumours were located on chronically sun-exposed sites, that is head and neck, in contrast to MMs, of which only 16% were located on the head and neck. More NHL cases than controls had a history of hospitalisation for a chronic inflammatory disease, such as a connective tissue disease, inflammatory bowel disease, and allergy, whereas previous history of these conditions was similar among skin cancer cases and their controls.

Use of oral glucocorticoids was slightly more frequent among BCC, SCC, MM, and NHL cases compared with controls. Adjusted IRRs were 1.15 (95% CI: 1.07–1.25) for BCC, 1.14 (95% CI: 0.94–1.39) for SCC, 1.15 (95% CI: 0.94–1.41) for MM, and 1.11 (95% CI: 0.85–1.46) for NHL (Table 2). For NHL, the risk increased linearly with the amount of glucocorticoids prescribed (IRR risk increase per 10 000 mg=2.26 (95% CI: 1.20–4.26), *P*=0.01). The restricted cubic spline showed the best fit to the data for SCC, and we found a statistically significant increased risk of SCC with increasing amounts of glucocorticoids prescribed (cumulative IRR per 10 000 mg=7.40 (95% CI: 1.80–30.4), *P*=0.001, data not shown). We found no dose–response relationship between glucocorticoid use and risk of BCC and MM for either the linear or the spline model (Table 2).

We found a slight trend of increasing risk of BCC among the users of glucocorticoids with longer duration of use, with IRRs increasing to 1.17 (95% CI: 1.08–1.28) among recorded users of more than 1 year, and to 1.22 (95% CI: 1.09–1.36) among recorded users of more than 5 years before the diagnosis date (Table 2). This pattern was not seen for SCC, MM, or NHL.

Among glucocorticoid users, the risk of a BCC located on the head and neck was slightly higher (IRR 1.23 (95% CI: 1.11–1.37)) than the risk of BCC at other anatomic sites (IRR 1.06 (95% CI: 0.94–1.20)). Such pattern was not seen for SCC and MM (data not shown).

The sensitivity analyses showed that if we had corrected for a differential under-ascertainment of NMSC cases from 5 to 45% (trapezoidal distribution) among users of glucocorticoids, the estimates for NMSC risk should have increased by a factor of 1.3 (Table 3).

## Discussion

We found a positive association between use of oral glucocorticoids and risk of BCC. The risk of BCC increased slightly with duration of use and was higher for tumours located on chronic sun-exposed areas, which support a causal explanation for our findings. We further detected a dose-related trend of increasing risk of SCC and NHL with increasing amount of glucocorticoid prescribed. However, these dose–response-related risk estimates had very limited statistical precision because they were based on low proportions of cases and should therefore be interpreted with caution. Our findings agree with the result of an earlier study in North Jutland showing a positive association between number of prescriptions for oral glucocorticoids and risk of BCC, SCC, and NHL (Sorensen *et al*, 2004). However, we did not find the same convincing association between oral glucocorticoid use, and risk of SCC and NHL (Sorensen *et al*, 2004). A possible explanation for this discrepancy may be that we excluded persons with prior cancers and organ transplants from this analysis. We also adjusted for other medical conditions and medications that may have confounded the results of the earlier study (Sorensen *et al*, 2004).

Our study has several strengths. The uniformly organised Danish health care system allows a truly population-based design. Data on oral glucocorticoids were collected prospectively and independent of the cancer data, limiting the potential for recall bias found in interview-based studies (Karagas, 1993). Moreover, by excluding glucocorticoid use within 1 and 5 years of the cancer diagnosis, we were able to assess the potential effect of a surveillance bias on our results. This surveillance bias refers to the possibility that users of oral glucocorticoids are under closer surveillance and probably receive more medical attention than non-users, and thus are expected to have a greater likelihood of having a cancer diagnosed. We found this to be a slight bias.

Among the limitations of our study is the use of data on filled prescription as a surrogate for actual use of the drugs. This could lead to misclassification of some non-users as users (non-adherence to the drug), leading to conservative risk estimates. However, the users had redeemed and paid for the prescription, and therefore this bias is likely to be small. Moreover, we may have under-adjusted for the chronic medical conditions that we considered to be confounding factors. Many patients suffering from these conditions are never referred to hospitals but are followed by their general practitioner. Another source of bias is left truncation of data from the Danish National Registry of Patients, which only dates back to 1977, and the Prescription Database, to 1989. Therefore, we may have missed some information on chronic diseases as confounding factors and exposures to oral glucocorticoids. This misclassification would likely be differential among cases and controls, as we would expect current users of oral glucocorticoids to have had more chronic diseases in the past, and a greater likelihood of earlier prescriptions of oral glucocorticoids. Thus, the resulting bias could lead us to either over- or underestimate our risk estimates. Another source of potential bias is that records of NMSC in particular may be unreported to the DCR (Frentz, 1996; Frentz and Olsen, 1999). As NMSC is rarely fatal, in severely diseased patients, such as glucocorticoid users, clinicians could potentially deem the NMSC trivial, and therefore pay less medical attention to them. As shown by the sensitivity analyses, this potential under-ascertainment of NMSC cases using glucocorticoids in our case–control study could have led to a substantial underestimation of the overall effect of glucocorticoid use on the risk of SCC and BCC.

Data such as skin phenotype (i.e., skin sensitivity to sunlight and sun exposure) and sun bathing habits were unavailable in this registry-based study. In addition, there may be some residual confounding from history of chronic diseases, as we were unable to adjust for the inflammatory activity (i.e., severity) of the individual chronic diseases. Consequently, we could not address these factors and other potential confounding effects on skin cancer, and NHL risk among patients taking oral glucocorticoids. The mechanisms behind our findings are not entirely clear, but our results support the hypothesis that immunosuppression by oral glucocorticoids is a risk factor for BCC. Our results are unlikely to be explained by confounding by the presence of chronic diseases or use of other immunosuppressants in conjunction with glucocorticoids.

## Figures and Tables

**Table 1:** Characteristics among cases with basal cell carcinoma, squamous cell carcinoma, malignant melanoma, and non-Hodgkin's lymphoma and controls (restricted to those cases with no history of organ transplants or cancer before the actual cancer under study)

	**Basal cell carcinoma**		**Squamous cell carcinoma**		**Malignant melanoma**		**Non-Hodgkin's lymphoma**	
	**Cases (*N*=5422)**	**Controls (*N*=22457)**	**Cases (*N*=935)**	**Controls (*N*=4166)**	**Cases (*N*=983)**	**Controls (*N*=4406)**	**Cases (*N*=481)**	**Controls (*N*=2004)**
*Demographic characteristics*								
Age (median)	68	68	77	77	58	58	64	64
Gender: male	50%	50%	67%	68%	41%	42%	53%	54%
								
*Anatomic site of skin cancer*								
Head and neck	54%	—	64%	—	16%	—	—	
Other sites	46%	—	36%	—	84%	—	—	
								
*Comorbidities/history of hospitalisation*								
Chronic pulmonary disease	299 (6%)	1234 (6%)	65 (7%)	306 (7%)	25 (3%)	187 (4%)	32 (7%)	100 (5%)
Connective tissue disease	123 (2%)	409 (2%)	33 (4%)	74 (2%)	16 (2%)	76 (2%)	13 (3%)	30 (2%)
Inflammatory bowel disease	33 (0.6%)	136 (0.6%)	5 (0.5%)	20 (0.5%)	5 (0.5%)	33 (0.8%)	5 (1%)	9 (0.5%)
Allergy	135 (2%)	432 (2%)	24 (3%)	91 (2%)	13 (1%)	90 (2%)	9 (2%)	27 (1%)
								
*Other drug use/prior drug prescriptions:*								
Methotrexate	30 (0.6%)	69 (0.3%)	2 (0.2%)	11 (0.2%)	4 (0.4%)	16 (0.4%)	2 (0.4%)	5 (0.3%)
Azathioprine	17 (0.3%)	57 (0.3%)	10 (1%)	8 (0.2%)	1 (0.1%)	9 (0.2%)	1 (0.2%)	3 (0.2%)

**Table 2:** Use of glucocorticoids and risk of skin cancer and non-Hodgkin's lymphoma

	**All prescriptions before diagnosis date**					**Prescriptions>1 year before diagnosis date**	**Prescriptions>5 years before diagnosis date**
	**Cases *N* (%)**	**Controls *N* (%)**	**IRR age and gender adjusted (95% CI)**	**IRR adjusted^a^ (95% CI)**	***P*-value for trend test (adjusted)**	**IRR adjusted^a^ (95% CI)**	**IRR adjusted^a^ (95% CI)**
*Basal cell carcinoma*							
No prescriptions	4300 (79%)	18278 (81%)	1.00	1.00	—	1.00	1.00
Any prescription	1122 (21%)	4179 (19%)	1.17 (1.08–1.27)	1.15 (1.07–1.25)		1.17 (1.08–1.28)	1.22 (1.09–1.36)
Risk increase per 10 000 mg^b^			1.10 (0.98–1.22)	1.07 (0.96–1.20)	0.2		
							
*Squamous cell carcinoma*							
No prescriptions	732 (78%)	3379 (81%)	1.00	1.00	—	1.00	1.00
Any prescription	203 (22%)	787 (19%)	1.21 (1.01–1.46)	1.14 (0.94–1.39)		1.09 (0.88–1.34)	1.11 (0.85–1.45)
Risk increase per 10 000 mg^b^			1.23 (0.98–1.54)	1.12 (0.88–1.43)^c^	0.4		
							
*Malignant melanoma*							
No prescriptions	820 (83%)	3696 (84%)	1.00	1.00	—	1.00	1.00
Any prescription	163 (17%)	710 (16%)	1.09 (0.90–1.33)	1.15 (0.94–1.41)		1.12 (0.90–1.39)	1.00 (0.75–1.36)
Risk increase per 10 000 mg^b^			1.11 (0.91–1.37)	0.94 (0.65–1.37)	0.8		
							
*Non-Hodgkin's lymphoma*							
No prescriptions	383 (80%)	1648 (82%)	1.00	1.00	—	1.00	1.00
Any prescription	98 (20%)	356 (18%)	1.20 (0.92–1.56)	1.11 (0.85–1.46)		1.08 (0.81–1.44)	1.04 (0.71–1.54)
Risk increase per 10 000 mg^b^			2.51 (1.41–4.49)	2.26 (1.20–4.26)	0.01		

CI=confidence interval; IRR=incidence rate ratio; SCC=squamous cell carcinoma.

a Conditional logistic regression was used to estimate IRRs and 95% CI, adjustments were made for a prior hospitalisation for selected chronic diseases and use of methotrexate and azathioprine.

b In this model we assumed a linear effect of the exposure.

c The restricted cubic spline showed the best fit to the data (Harre *et al*, 1988), with a statistically significant increased risk of SCC with increasing amounts of glucocorticoids prescribed (cumulative IRR per 10 000 mg=7.40 (95% CI: 1.80–30.4), *P*=0.001).

**Table 3:** Probabilistic sensitivity analyses correcting for differential non-reporting of cases with NMSC with sensitivity and specificity drawn from trapezoidal and uniform distributions

**Analysis**	**Basal cell carcinoma^a^**			**Squamous cell carcinoma**		
	**IRR 2.5 percentile**	**IRR median estimate**	**IRR 97.5 percentile**	**IRR 2.5 percentile**	**IRR median estimate**	**IRR 97.5 percentile**
Conventional analysis	1.07	1.15	1.25	0.94	1.15	1.40
Differential sensitivity analysis–systematic error	1.17	1.45	1.92	1.15	1.47	2.00
Differential sensitivity analysis–systematic and random error	1.14	1.45	1.94	1.06	1.48	2.16

BCC=basal cell carcinoma; IRR=incidence rate ratios; NMSC=non-melanoma skin cancer.

Sensitivity among cases using glucocorticoids: Trapezodial distribution (minimum=0.55, mode 1=0.6, mode 2=0.9, maximum=0.95). Sensitivity among cases not using glucocorticoids: Uniform distribution (minimum=0.8, maximum=1.0). Specificity among non-cases: Uniform distribution (minimum=0.95, maximum=1.0).

a The sensitivity analysis should be cautiously interpreted for risk of BCC among oral glucocorticoid users, because the SAS-macro did not converge after approximately 25 000 iterations. Therefore, the macro was reran and stopped after 5000 iterations.

**Table A1:** The ATC codes for oral glucocorticoids

Glucocorticoids	ATC codes
Budenoside	A07EA06
Hydrocortisone	A07EA02, H02AB09
Prednisolone	A07EA01, H02AB06
Prednisone	H02AB07
Betamethasone	H02AB01
Methylprednisolone	H02AB04
Triamcinolone	H02AB08

ATC=anatomical therapeutical chemical.

**Table A2:** Classification of chronic diseases and ICD-8 and ICD-10 diagnoses codes

Disease category	Diseases	ICD-8	ICD-10
Chronic pulmonary disease	Emphysema and chronic obstructive lung disease	490–493; 515–518	J40–J47; J60–J67; J68.4; J70.1; J70.3; J84.1; J92.0; J96.1; J98.2; J98.3
Connective tissue disease	Diffuse connective tissue disease, Sjoegren's syndrome, rheumatoid arthritis and other inflammatory polyarthropathies and polymyalgia rheumatica	712; 716; 734; 446; 135.99	M05; M06; M08; M09; M30–M36; D86
Inflammatory	Colitis ulcerosa	561	K50
Bowel diseases	Crohn's disease	563	K51
Allergy	Atopic dermatitis	502; 493;	J30, J45–J47
	Asthma	692.59;	L20, K52.2
	Food allergy	691; 692	T78

## Appendix 1

Table A1

## Appendix 2

Table A2

## References

[bib1] Andersen TF, Madsen M, Jorgensen J, Mellemkjoer L, Olsen JH (1999) The Danish National Hospital Register. A valuable source of data for modern health sciences. Dan Med Bull 46: 263–268 10421985

[bib2] Askling J, Klareskog L, Hjalgrim H, Baecklund E, Bjorkholm M, Ekbom A (2005) Do steroids increase lymphoma risk? A case–control study of lymphoma risk in polymyalgia rheumatica/giant cell arteritis. Ann Rheum Dis 64: 1765–1768 15843445 10.1136/ard.2005.036459 PMC1755302

[bib3] Baecklund E, Iliadou A, Askling J, Ekbom A, Backlin C, Granath F, Catrina AI, Rosenquist R, Feltelius N, Sundstrom C, Klareskog L (2006) Association of chronic inflammation, not its treatment, with increased lymphoma risk in rheumatoid arthritis. Arthritis Rheum 54: 692–701 16508929 10.1002/art.21675

[bib4] Dukes MNG (1993) Drug Utilization Studies: Methods and Uses. WHO Regional Publications: Geneva 8442841

[bib5] Fox MP, Lash TL, Greenland S (2005a) A method to automate probabilistic sensitivity analyses of misclassified binary variables. Int J Epidemiol 34: 1370–1376 16172102 10.1093/ije/dyi184

[bib6] Fox MP, Lash TL, Greenland S (2005b) Boston University sensitivity analysis tools. Available at: http://sph.bu.edu/index.php?option=com_content&task=view&id=405&Itemid=508#epidemiologic

[bib7] Frank L (2000) Epidemiology. When an entire country is a cohort. Science 287: 2398–2399 10766613 10.1126/science.287.5462.2398

[bib8] Frentz G (1996) General skin cancer. Quantity, treatment and quality. Ugeskr Laeger 158: 7202 9012031

[bib9] Frentz G, Olsen JH (1999) Malignant tumours and psoriasis: a follow-up study. Br J Dermatol 140: 237–242 10233215 10.1046/j.1365-2133.1999.02655.x

[bib10] Gaist D, Sorensen HT, Hallas J (1997) The Danish prescription registries. Dan Med Bull 44: 445–448 9377907

[bib11] Glover MT, Deeks JJ, Raftery MJ, Cunningham J, Leigh IM (1997) Immunosuppression and risk of non-melanoma skin cancer in renal transplant recipients. Lancet 349: 398 10.1016/S0140-6736(97)80015-3 9033469

[bib12] Greenland S (2005) Multiple-bias modeling for analysis of observational data (with discussion). J R Stat Soc 168: 267–306

[bib13] Harre FE, Lee KL, Pollock BG (1988) Regression models in clinical studies: determining relationships between predictors and response. J Natl Cancer Inst 80: 1198–1202 3047407 10.1093/jnci/80.15.1198

[bib14] Jensen AO, Olesen AB, Dethlefsen C, Sorensen HT (2007) Do incident and new subsequent cases of non-melanoma skin cancer registered in a Danish prospective cohort study have different 10-year mortality? Cancer Detect Prev 31: 352–358 18031945 10.1016/j.cdp.2007.04.011

[bib15] Jensen AO, Olesen AB, Dethlefsen C, Sorensen HT, Karagas MR (2008) Chronic diseases requiring hospitalization and risk of non-melanoma skin cancers – a population based study from Denmark. J Invest Dermatol 128: 926–931 17914446 10.1038/sj.jid.5701094

[bib16] Jensen P, Hansen S, Moller B, Leivestad T, Pfeffer P, Geiran O, Fauchald P, Simonsen S (1999) Skin cancer in kidney and heart transplant recipients and different long-term immunosuppressive therapy regimens. J Am Acad Dermatol 40: 177–186 10025742 10.1016/s0190-9622(99)70185-4

[bib17] Karagas MR (1993) Are administratively collected data useful for case–control studies? Ann Epidemiol 3: 111–112 8287145 10.1016/1047-2797(93)90018-y

[bib18] Karagas MR, Cushing Jr GL, Greenberg ER, Mott LA, Spencer SK, Nierenberg DW (2001) Non-melanoma skin cancers and glucocorticoid therapy. Br J Cancer 85: 683–686 11531252 10.1054/bjoc.2001.1931 PMC2364134

[bib19] Kinlen LJ (1992) Malignancy in autoimmune diseases. J Autoimmun 5(Suppl A): 363–371 10.1016/0896-8411(92)90055-u 1503633

[bib20] Le Vay D, Loxton GE (1949) Deoxycortone acetate and ascorbic acid in the treatment of rheumatoid arthritis. Lancet 2: 1134 15396965 10.1016/s0140-6736(49)91149-6

[bib21] Marcen R, Pascual J, Tato AM, Teruel JL, Villafruela JJ, Fernandez M, Tenorio M, Burgos FJ, Ortuno J (2003) Influence of immunosuppression on the prevalence of cancer after kidney transplantation. Transplant Proc 35: 1714–1716 12962768 10.1016/s0041-1345(03)00669-9

[bib22] Nielsen GL, Sorensen HT, Weijin Z, Steffensen FH, Olsen J (1997) The Pharmacoepidemiologic Prescription Database of North Jutland – a valid tool in pharmacoepidemiological research. Int J Risk Saf Med 10: 203–205 23511375 10.3233/JRS-1997-10309

[bib23] Rothman KJ (2002) Epidemiology: An Introduction. Oxford University Press: New York

[bib24] Sorensen HT, Christensen S, Mehnert F, Pedersen L, Chapurlat RD, Cummings SR, Baron JA (2008) Use of bisphosphonates among women and risk of atrial fibrillation and flutter: population based case–control study. BMJ 336: 813–816 18334527 10.1136/bmj.39507.551644.BE PMC2292333

[bib25] Sorensen HT, Mellemkjaer L, Nielsen GL, Baron JA, Olsen JH, Karagas MR (2004) Skin cancers and non-Hodgkin lymphoma among users of systemic glucocorticoids: a population-based cohort study. J Natl Cancer Inst 96: 709–711 15126608 10.1093/jnci/djh118

[bib26] Storm HH, Michelsen EV, Clemmensen IH, Pihl J (1997) The Danish Cancer Registry – history, content, quality and use. Dan Med Bull 44: 535–539 9408738

[bib27] Szklo M, Nieto FJ (2000) Epidemiology: Beyond the Basics. Aspen: Gaithersburg, MD

[bib28] Wacholder S, McLaughlin JK, Silverman DT, Mandel JS (1992) Selection of controls in case–control studies. I. Principles. Am J Epidemiol 135: 1019–1028 1595688 10.1093/oxfordjournals.aje.a116396

[bib29] Zoorob RJ, Cender D (1998) A different look at corticosteroids. Am Fam Physician 58: 443–450 9713398

